# CHFR regulates chemoresistance in triple-negative breast cancer through destabilizing ZEB1

**DOI:** 10.1038/s41419-021-04114-8

**Published:** 2021-08-30

**Authors:** Hong Luo, Zhicheng Zhou, Shan Huang, Mengru Ma, Manyu Zhao, Lixu Tang, Yuan Quan, Yiming Zeng, Li Su, Jongchan Kim, Peijing Zhang

**Affiliations:** 1 National Engineering Research Center for Nanomedicine, Key Laboratory of Molecular Biophysics of Ministry of Education, College of Life Science and Technology, Tongji Hospital, Huazhong University of Science and Technology, 430074 Wuhan, China; 2 Department of Experimental Radiation Oncology, The University of Texas MD Anderson Cancer Center, Houston, TX 77030 USA; 3 School of Martial Arts, Wuhan Sports University, 430079 Wuhan, China; 4 Stem Cell Laboratory, the Second Affiliated Hospital, Fujian Medical University, 362000 Quanzhou, China; 5 Department of Life Science, Sogang University, Seoul, 04107 Republic of Korea

**Keywords:** Cancer therapy, Cell death, Post-translational modifications

## Abstract

Failures to treat triple-negative breast cancer (TNBC) are mainly due to chemoresistance or radioresistance. We and others previously discovered that zinc finger E-box-binding homeobox 1 (ZEB1) is a massive driver causing these resistance. However, how to dynamically modulate the intrinsic expression of ZEB1 during cell cycle progression is elusive. Here integrated affinity purification combined with mass spectrometry and TCGA analysis identify a cell cycle-related E3 ubiquitin ligase, checkpoint with forkhead and ring finger domains (CHFR), as a key negative regulator of ZEB1 in TNBC. Functional studies reveal that CHFR associates with and decreases ZEB1 expression in a ubiquitinating-dependent manner and that CHFR represses fatty acid synthase (FASN) expression through ZEB1, leading to significant cell death of TNBC under chemotherapy. Intriguingly, a small-molecule inhibitor of HDAC under clinical trial, Trichostatin A (TSA), increases the expression of CHFR independent of histone acetylation, thereby destabilizes ZEB1 and sensitizes the resistant TNBC cells to conventional chemotherapy. In patients with basal-like breast cancers, CHFR levels significantly correlates with survival. These findings suggest the therapeutic potential for targeting CHFR-ZEB1 signaling in resistant malignant breast cancers.

## Introduction

Malignant breast cancers such as triple-negative breast cancer (TNBC) often cause clinical drug resistance, and there is no suitable drug targets [1, 2]. The combination of drugs has been proven to eliminate the resistant cancer [3]. Zinc finger E-box-binding homeobox 1 (ZEB1) protein is a key transcriptional factor that induces epithelial–mesenchymal transition and cellular plasticity in cancer cells [4–8]. ZEB1 has also been reported to transform non-invasive breast cancer cells into highly malignant cancer stem cells (CSCs) [9, 10]. Moreover, we and others recently demonstrate that ZEB1 protein can promote the resistance of breast cancer to radiotherapy and chemotherapy [10–14]. Together, ZEB1 is a massive driver of breast cancers causing death, although the regulatory mechanisms of highly expressed ZEB1 in breast cancers remain much less understood. Previously, we found that ZEB1 is dynamically regulated in the cell cycle progression. ZEB1 protein is dramatically decreased from G2/M phase to G1 phase without changing mRNA level [12]. However, the inactivating signals or regulators that make ZEB1 protein quickly disappear remain unclear. In this study, we conduct an integrated analysis to identify a ZEB1-binding E3 ubiquitin ligase, checkpoint with forkhead and ring finger domains (CHFR).

It is established that CHFR is silenced in many primary cancers including breast cancers due to CpG methylation and histone deacetylation [15, 16]. Consistent with its expression, CHFR plays an important role in tumor suppression through blocking cell cycle progression or inhibiting metastasis [17–19]. However, it is not clear whether CHFR expression will benefit the treatment for resistant breast cancers. Given that CHFR is highly methylated and silenced in G2/M phase in gastric cancer [16], it is speculated that CHFR is an E3 ubiquitin ligase for ZEB1 and exerts its tumor suppression through destabilizing ZEB1 in TNBC.

In this work, we discovered that small-molecule inhibitor of histone deacetylase (HDAC), Trichostatin A (TSA), significantly enhanced the expression of CHFR protein though directly binding to CHFR, which is independent of HDAC enzymatic activity. More importantly, by targeting CHFR-ZEB1 signaling, these findings define a previously unknown function of CHFR and establish a potential therapeutic strategy through combination of TSA with chemotherapeutic drugs such as doxorubicin (DOX), paclitaxel, or fluorouracil (5-FU) for TNBC patients.

## Results

### CHFR interacts and negatively correlates with ZEB1

**Fig. 1:**
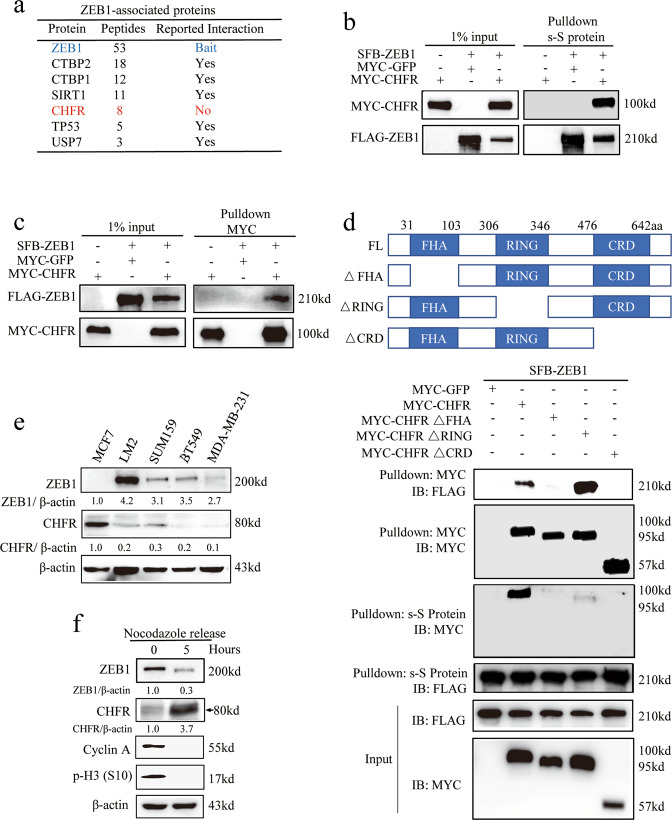
CHFR interacts and negatively correlates with ZEB1. **a** Mass spectrometry identified a partial list of ZEB1 associated proteins. **b**, **c** Transiently transfected SFB-ZEB1, MYC-CHFR, and MYC-GFP into HEK293T cells, followed by pulldown with Sepharose beads (s-S beads) (**b**) or MYC beads (**c**) and immunoblotting with the antibodies indicated. **d** The schematic diagram of FHA, RING, and CRD domains of CHFR. Full-length CHFR (FL), CHFR∆FHA, CHFR∆RING, and CHFR∆CRD were transiently transfected into HEK293T cells, followed by pulldown with Sepharose beads (s-S beads) or MYC beads and immunoblotting with the antibodies indicated. **e** Immunoblotting of ZEB1, CHFR, and β-actin in non-TNBC cell MCF7 and TNBC cells LM2, SUM159, BT549, and MDA-MB-231. **f** SUM159 cells were treated with 0.5 µg ml^−1^ nocodazole overnight, the mitotic cells were “shaken off,” and then released into normal medium. Samples were collected at 0 and 5 h after releasing and analyzed by western blotting with ZEB1, CHFR, and β-actin. Cell cycle distribution was marked by Cyclin A and p-H3 (S10).

We previously found that ZEB1 is highly expressed in breast cancers [12] and confers basal breast cancer resistant to conventional chemotherapy and neoadjuvant therapy (Fig. S1a–d). More importantly, ZEB1 protein is dynamically changed during cell cycle progression [12], which is negatively correlated with the sensitivity of cancer cells to chemotherapeutic treatment (Fig. S1e). As shown, when synchronized SUM159 TNBC cells in the G2/M phase by nocodazole treatment and released at different time points, cells in G2/M phase (0 h after nocodazole release) with higher ZEB1 expression is more resistant to doxorubicin treatment compared to the cells in G1 phase (5 h after nocodazole release) without ZEB1 (Fig. S1e). To further investigate how ZEB1 is dynamically regulated, we set out to identify new ZEB1-interacting proteins using a triple-epitope (S-protein, FLAG tag, and streptavidin-binding peptide)-tagged version of ZEB1 (SFB-ZEB1). Tandem-affinity purification using streptavidin Sepharose beads and S-protein agarose beads followed by mass spectrometric analysis identified several reported ZEB1 interactors, including USP7, p53, CTBP2, CTBP1, and SIRT1 [12, 20–22], as well as a previously undescribed ZEB1 interactor, CHFR (Fig. 1a and Supplementary Table 1). Co-immunoprecipitation assays confirmed that CHFR could be detected in ZEB1 immunoprecipitates (Fig. 1b) and that ZEB1 were present in CHFR immunoprecipitates (Fig. 1c). We further mapped the domain of CHFR required for its interaction with ZEB1. MYC-tagged truncates of CHFR were generated (Fig. 1d, upper panel) and co-expressed with FLAG-ZEB1. Western blot analysis of MYC immunoprecipitates revealed that both the forkhead-associated (FHA) domain and carboxy-terminal cysteine-rich (CR) region of CHFR are necessary for the interaction with ZEB1, while the RING domain is dispensable (Fig. 1d, lower panel). Also, we transfected ZEB1 truncates described previously [23] with wild-type CHFR into HEK293T cells and found that only ZEB1 N-terminal zinc finger domain (NZF) interacts with CHFR (Fig. S2a).

To determine whether ZEB1 indeed correlate with CHFR in TNBC cells since CHFR has been reported as a cell cycle regulatory E3 ligase with several reported substrates [24–27], we performed immunoblotting analysis of these two proteins in human TNBC and non-TNBC cell lines. Consistently, ZEB1 is highly expressed in TNBC cells, while CHFR is not or lowly expressed. Interestingly, a significant negative correlation between CHFR and ZEB1 was observed in these breast cancer cells, in which most of the tumors with high ZEB1 expression exhibited low CHFR expression (Fig. 1e). We also plotted the relative CHFR expression score versus the ZEB1 expression score for individual patients from The Cancer Genome Atlas (TCGA) database, which revealed a highly negative correlation (linear regression *R*^2^ = 0.94, Fig. S1f). These results indicates that downregulation of CHFR may contribute to overexpression of ZEB1 in human TNBC patients, which may lead to drug resistance and eventually relapse.

Moreover, when synchronized SUM159 cells in the G2/M phase by nocodazole treatment and released the cells for 5 h, we found that, in these synchronized cells, ZEB1 levels negatively correlated with CHFR levels, which suggested that the dynamic regulation of ZEB1 may be a direct effect of CHFR. Taken together, as a cell cycle regulatory E3 ligase, CHFR may contribute to the dysregulation of ZEB1 and the relative drug resistance of TNBC.

### CHFR is a ZEB1 E3 ligase

**Fig. 2:**
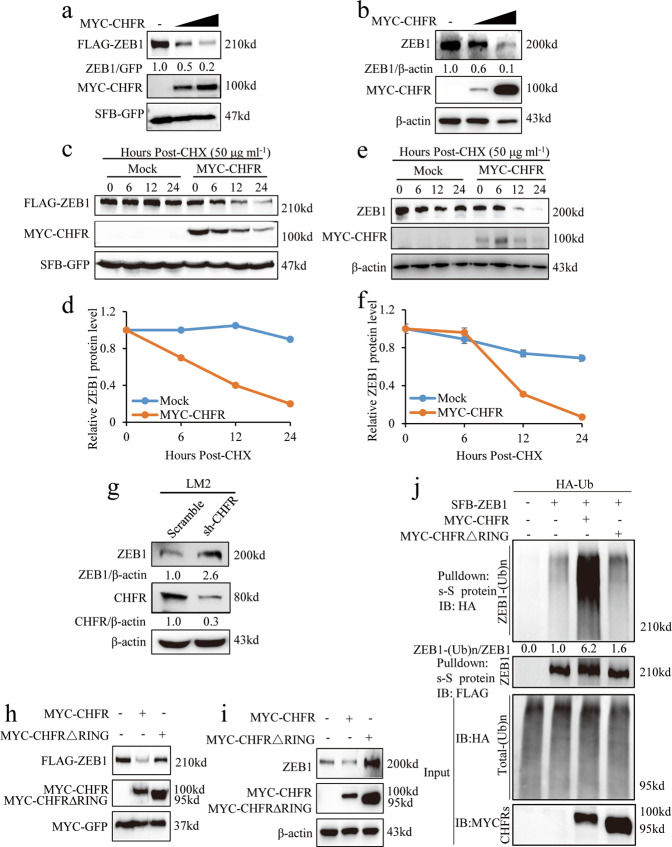
CHFR is a ZEB1 E3 ligase. **a** Transiently transfected SFB-ZEB1 and different concentrations of MYC-CHFR in HEK293T cells and immunoblotted with the MYC and FLAG antibodies. **b** In SUM159 cells, different concentrations of MYC-CHFR were transfected, followed by immunoblotting with the antibodies as indicated. **c**, **d** SFB-ZEB1 and MYC-CHFR were transiently transfected into HEK293T cells, and 50 μg ml^−1^ CHX was added, and samples were collected at the indicated time points and analyzed by western blotting with FLAG and MYC antibodies. **e**, **f** In SUM159 stably expressed mock or CHFR cells, 50 μg ml^−1^ CHX was added, and samples were collected at the indicated time points, followed by immunoblotting with antibodies against ZEB1, MYC, and β-actin. **g** LM2 was transduced by scramble or sh-CHFR cells, and samples were collected and immunoblotted by ZEB1, CHFR, and β-actin antibodies. **h** Transiently transferred SFB-ZEB1, MYC-CHFR, or MYC-CHFR∆RING into HEK293T cells. Immunoblotting of MYC and FLAG. **i** Transiently transfected MYC-CHFR or MYC-CHFR∆RING into SUM159 cells, followed by immunoblotting of ZEB1, MYC, and β-actin. **j** HEK293T cells were transiently transfected with SFB-ZEB1, MYC-CHFR, or MYC-CHFR ∆RING and added MG132 for 6 h before collecting samples, followed by pulling down with s-S protein beads and immunoblotting of MYC, HA, and FLAG.

To determine whether CHFR affect ZEB1 protein levels, we co-transfected ZEB1 and CHFR into HEK293T cells and found that CHFR downregulated ectopically expressed ZEB1 protein level in a dose-dependent manner (Fig. 2a). Consistently, overexpression of CHFR significantly inhibited endogenous ZEB1 protein level in SUM159 cells (Fig. 2b). In addition, CHFR only inhibit the expression of the ZEB1 mutants containing NZF domain, further supporting that CHFR specifically associates with and regulates ZEB1 protein (Fig. S2b). To determine whether CHFR destabilizes ZEB1 protein, we first examined the levels of ectopically expressed ZEB1 protein in HEK293T cells in the presence of cycloheximide (CHX), an inhibitor of protein synthesis. As expected, overexpression of CHFR led to a prominent decrease in the half-life of ZEB1 protein, whereas the level and stability of co-transfected SFB-GFP were not affected (Fig. 2c, d). Also, compared to control cells, in SUM159 cells that stably expressed CHFR, endogenous ZEB1’s half-life is dramatically shortened (Fig. 2e, f). Conversely, knockdown of CHFR in LM2 TNBC cells markedly increase the ZEB1 expression level (Fig. 2g). Given that RING domain is responsible for CHFR activity, we co-transfected ZEB1 together with wild-type CHFR and RING domain deletion mutant (dead mutant) into HEK293T cells. As expected, wild-type CHFR can degrade the ectopically expressed ZEB1, while the dead mutants cannot (Fig. 2h). Consistently, the RING domain deletion mutant cannot degrade the endogenous ZEB1 protein in SUM159 cells compared to wild-type CHFR (Fig. 2i). We reasoned that CHFR destabilized ZEB1 through proteasome-dependent ubiquitination. Indeed, overexpressed wild-type CHFR substantially increase the ubiquitination of ZEB1, while the dead mutant has no effect on ubiquitination of ZEB1 (Fig. 2j). Moreover, molecular docking analysis were performed using AutoDock Vina (Version 1.1.2) [28] and suggested a favorable binding mode between the region of ZEB1 and the FHA/CRD domain of CHFR (Fig. S2c). We concluded from these experiments that ZEB1 is a substrate of CHFR and that CHFR directly interacts with and degrades ZEB1 in the ubiquitin-dependent manner.

### CHFR regulates chemosensitivity in TNBC cells

**Fig. 3:**
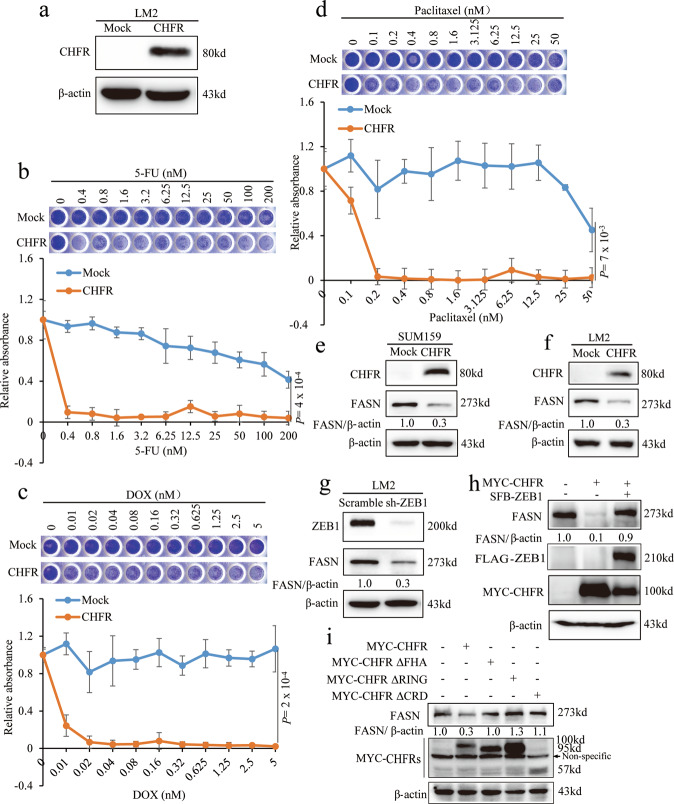
CHFR regulates chemosensitivity in TNBC cells. **a** CHFR was stably transduced in LM2 cells and immunoblotted with the CHFR and β-actin antibodies. **b**–**d** LM2 cells stably transformed with mock or CHFR were seeded in 96-well plates and treated with 5-FU, DOX, or paclitaxel as indicated and then fixed with 10% methanol and stained with 1:1000 crystal violet. **e**, **f** CHFR was stably transduced in SUM159 and LM2 cells and immunoblotted with the CHFR, FASN, and β-actin antibodies. **g** ZEB1 was stably knocked down in LM2 cells and immunoblotted with the ZEB1, FASN, and β-actin antibodies. **h** CHFR and ZEB1 were transfected as indicated and immunoblotted with the MYC, FLAG, FASN, and β-actin antibodies. **i** Full-length MYC-CHFR and MYC-CHFR∆FHA, MYC-CHFR∆RING, and MYC-CHFR∆CRD mutants were transiently transfected in SUM159 cells and immunoblotted with the MYC, FASN and β-actin antibodies. Significance of Mock versus CHFR is shown. *n* = 3 wells per group. Data in **b**–**d** are the mean of biological replicates from a representative experiment, and error bars indicate s.e.m. Statistical significance was determined by a two-tailed, unpaired Student’s *t* test. The experiments were repeated three times.

Whereas ZEB1 has been shown to confer different types of cancer drug resistance including in breast cancer, the function of CHFR in chemoresistance of TNBC cells remains unclear. Since endogenous CHFR expression in LM2 is not high, we made stably overexpressed CHFR cell line (Fig. 3a) and analyzed the cell proliferation. As expected, compared to control cells, CHFR overexpression substantially sensitized LM2 cells to conventional chemotherapeutic agents such as 5-FU (Figs. 3b and S3a), doxorubicin (Fig. 3c), and paclitaxel (Fig. 3d). Similarly, for other TNBC cells such as BT549 and SUM159 cells, ectopically expressed CHFR can dramatically promote cell death under 5-FU treatment (Fig. S3b, c). As a key lipogenic enzyme catalyzing the terminal steps in the de novo biogenesis of fatty acids, fatty acid synthase (FASN) has been established to confer malignant tumor growth and survival [29]. Recently, preclinical evaluation showed that FASN is overexpressed in TNBC tumor samples and contributes to doxorubicin [30] and docetaxel resistance [31]. To assess the effect of CHFR on FASN, we stably transduced CHFR in SUM159 and LM2 cells. Clearly, we found that CHFR overexpression significantly inhibit FASN protein level in both TNBC cells (Fig. 3e, f). Moreover, knockdown of ZEB1 could markedly downregulate FASN expression in LM2 cells (Fig. 3g). To further assess whether the effect of CHFR on FASN through ZEB1, we overexpressed ZEB1 to see whether it can rescue FASN levels in CHFR-expressing cells, as expectedly, CHFR does inhibit FASN expression though ZEB1 protein (Fig. 3h). Notably, overexpression of wild-type CHFR into SUM159 cells could nearly abolish the endogenous FASN protein but not the non-interactive mutants (∆FHA, ∆CRD) and dead mutant (Fig. 3i). Collectively, CHFR regulates FASN protein level through interacting with and destabilizing ZEB1 and further weakens the chemoresistance of TNBC cells.

### TSA treatment downregulates ZEB1 protein level through stabilizing CHFR

**Fig. 4:**
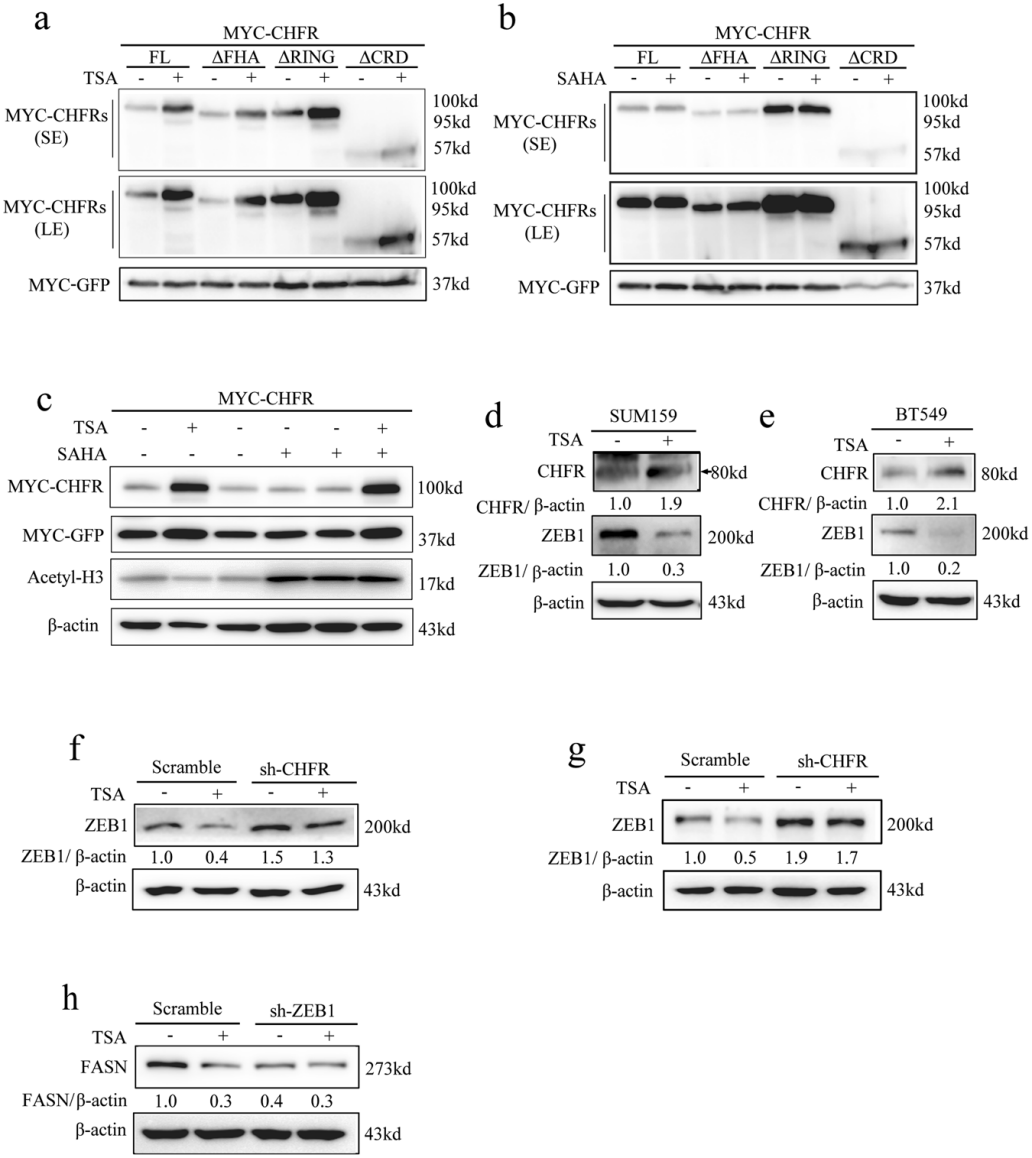
TSA treatment downregulates ZEB1 protein level through stabilizing CHFR. **a**, **b** Full-length MYC-CHFR, MYC-CHFR∆FHA, MYC-CHFR∆RING, MYC-CHFR∆CRD, and MYC-GFP were transiently transfected into HEK293T cells and then treated with 1 μM TSA for 36 h and immunoblotted with the MYC antibody. SE: short exposure, LE: long exposure (**a**); cells were also treated with 1 μM SAHA for 36 h and immunoblotted with the MYC antibody (**b**). **c** MYC-CHFR was transfected into HEK293T cells, pretreated with or without SAHA for 6 h, treated with 1 μM TSA or SAHA as indicated for 36 h, and then immunoblotted with the MYC, Acetyl-H3, and β-actin antibodies. **d**, **e** SUM159 and BT549 cells were treated with 1 μM TSA for 36 h and immunoblotted with the CHFR, ZEB1, and β-actin antibodies. **f**, **g** LM2 and BT549 cells that stably knocked down CHFR were treated with 1 μM TSA for 36 h and immunoblotted with the ZEB1 and β-actin antibodies. **h** LM2 cells that stably knocked down ZEB1 were treated with 1 μM TSA for 36 h and immunoblotted with the FASN and β-actin antibodies.

Given that CHFR is low expressed in TNBC cells, we sought to search small molecules that could boost the endogenous CHFR and further inhibit ZEB1 protein level. As it was reported that combination of both TSA and 5-aza-2’-deoxycytidine (5-aza-dC) reactivate the transcription of CHFR in RKO colorectal cancer cells [15], we assessed the effect of those agents on CHFR mRNA level in TNBC cells. Accordingly, we treated the 5 TNBC cell lines as indicated with individual compound such as TSA or 5-aza-dC. Unfortunately, none of them regulate the mRNA level of CHFR (Fig. S4a and data not shown). However, TSA treatment markedly enhanced the CHFR protein level in MDA-MB-231 cells (Fig. S4b), but not 5-aza-dC (data not shown). Similarly, when co-transfected MYC-tagged wild type and the truncates as indicated into HEK293T cells, we found that both full-length CHFR and the other truncates such as ∆FHA, ∆CRD, and ∆RING have been strongly upregulated by TSA treatment (Fig. 4a). Since TSA is a HDAC inhibitor, we therefore wanted to confirm whether CHFR regulated by TSA is histone acetylation dependent. SAHA, another potent inhibitor of HDAC [32], was used to treat the co-transfected HEK293T cells as described in Fig. 4a. Strikingly, we found that SAHA treatment could not change the expression level of wild-type CHFR and the relative truncates (Fig. 4b). Moreover, we ectopically overexpressed CHFR in HEK293T cells and pretreated with SAHA for 6 h, after that, cells were treated with or without TSA for additional 30 h. We found that TSA still strongly upregulates the CHFR protein level, although the activity of HDACs was markedly blocked by SAHA (Fig. 4c). We conclude from those experiments that, unlike Toyota’s finding [15], we found that TSA treatment dramatically induce the CHFR protein expression in TNBC cells, which is histone acetylation independent. The detailed mechanisms warrants future investigation. To further assess whether TSA treatment could downregulate ZEB1 protein level, we treated SUM159 and BT549 TNBC cells with TSA. Consistently, TSA treatment increases the endogenous CHFR protein level and, as expectedly, drastically decreases the endogenous ZEB1 protein level (Fig. 4d, e). To determine whether the effect of TSA on ZEB1 expression is mediated by CHFR, we transduced short hairpin RNA (shRNA) for CHFR in LM2 and BT549 cells and found that knockdown of CHFR almost completely reverses the effect of TSA as shown in scramble (Fig. 4f, g). We also found that TSA treatment leads to a decrease of FASN protein level in scramble compared to the sh-ZEB1 groups (Fig. 4h). Collectively, our data suggest that TSA is a functional activator for CHFR and an effective scavenger for ZEB1 in TNBC cells.

### TSA treatments sensitizes the TNBC cells to chemotherapeutic agents

**Fig. 5:**
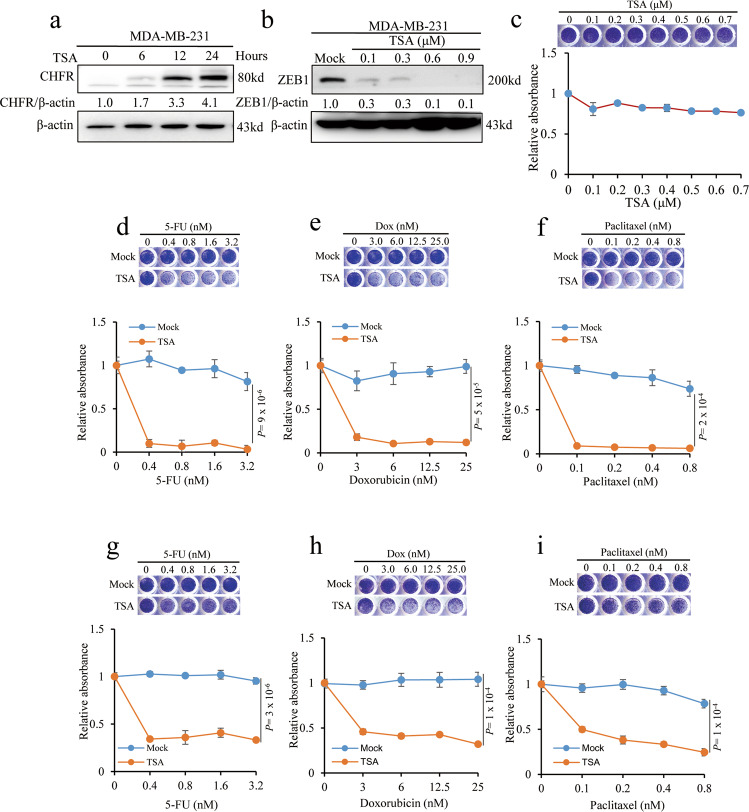
TSA treatments sensitizes the TNBC cells to chemotherapeutic agents. **a** MDA-MB-231 cells were treated with 1 μM TSA for hours as indicated and immunoblotted with the CHFR and β-actin antibodies. **b** MDA-MB-231 cells were treated with different concentrations of TSA for 36 h and immunoblotted with the ZEB1 and β-actin antibodies. **c** MDA-MB-231 cells were treated with TSA as indicated for 36 h. After fixing with 10% methanol, the cells were then stained with 1:1000 crystal violet. **d**–**f** MDA-MB-231 cells were co-treated with 0.6 μM TSA and 5-FU (**d**), DOX (**e**), or paclitaxel (**f**) for 36 h. After fixing with 10% methanol, the cells were then stained with 1:1000 crystal violet. **g**–**i** LM2 cells were treated and analyzed as described in **d**–**f**. Significance of Mock versus TSA is shown. *n* = 3 wells per group. Data in **c** and **d**–**i** are the mean of biological replicates from a representative experiment, and error bars indicate s.e.m. Statistical significance was determined by a two-tailed, unpaired Student’s *t* test. The experiments were repeated three times.

We asked whether TSA regulates CHFR/ZEB1 protein level and further inhibit cell viability. Consistently, treatment of MDA-MB-231 cells with TSA increases CHFR protein level in a time-dependent manner (Fig. 5a) and decreases ZEB1 protein in a dosage-dependent manner (Fig. 5b). However, individually treated with TSA has little effect on cell viability of MDA-MB-231, even at concentration of 0.7 μM (Fig. 5c). Interestingly, when co-treated with 5-FU, doxorubicin, and paclitaxel chemotherapeutic drugs, TSA could nearly eliminate the survival ability of MDA-MB-231 cells, whereas individual chemotherapy has almost no effect at high dosage (Fig. 5d–f). Similar effects were observed in LM2 cells (Fig. 5g–i). Taken together, these data suggest that the preclinical potential of TSA plus conventional chemotherapeutic drugs co-treatments could be applicable to conquer the resistant TNBC cells.

### TSA suppresses chemoresistance through CHFR

**Fig. 6:**
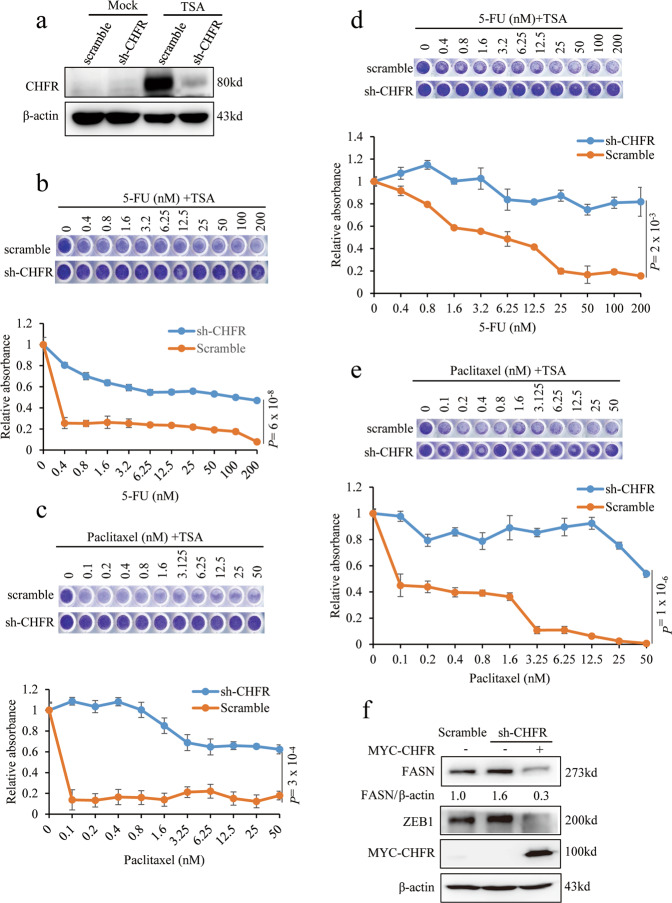
TSA suppresses chemoresistance through CHFR. **a** CHFR was stably knocked down in LM2 cells and treated with or without 0.6 μM TSA and immunoblotted with the CHFR and β-actin antibodies. **b**, **c** Scramble and sh-CHFR stable LM2 cells as described in **a** were co-treated with 0.6 μM TSA and the chemotherapy drugs 5-FU (**b**) or paclitaxel (**c**) for 36 h. After fixing with 10% methanol, the cells were then stained with 1:1000 crystal violet. **d**, **e** Scramble and sh-CHFR stable MDA-MB-231 cells were treated and analyzed as described in **b**, **c**. **f** LM2 cells that stably knocked down CHFR were rescued with or without MYC-CHFR and immunoblotted with the FASN, ZEB1, MYC, and β-actin antibodies. Significance of scramble versus sh-CHFR is shown. *n* = 3 wells per group. Data in **b**–**e** are the mean of biological replicates from a representative experiment, and error bars indicate s.e.m. Statistical significance was determined by a two-tailed, unpaired Student’s *t* test. The experiments were repeated three times.

We next sought to determine whether TSA treatments sensitizes the TNBC cells to chemotherapy by mediating stimulation of CHFR expression. We transduced shRNA for CHFR in LM2 cells and treated with TSA. Consistently, TSA treatment significantly induces CHFR expression in scramble cells; on the contrary, it almost does not promote CHFR expression in sh-CHFR stable cells (Fig. 6a). Intriguingly, the marked effect of co-treatment with TSA and 5-FU, doxorubicin, or paclitaxel relatively on LM2 cell viability in scramble cells is nearly completely abolished by knockdown of CHFR (Figs. 6b, c and S5a). Similarly, knockdown of CHFR in MDA-MB-231 cells strongly restore the resistance to the co-treatment between TSA and 5-FU, doxorubicin, or paclitaxel (Figs. 6d, e and S5b). To further determine whether the effect of TSA-CHFR is mediated by the downstream functional regulator, FASN, we restored CHFR expression in CHFR knockdown cells, and consistently, knock down of CHFR significantly upregulates FASN and ZEB1 protein level, which could be completely reversed by overexpression of CHFR (Fig. 6f). Collectively, our data strongly suggest that TSA promotes sensitizing of TNBC cells to chemotherapy mainly through CHFR-mediated repression of ZEB1 and FASN expression.

### CHFR promotes apoptosis and correlates with good clinical outcome in malignant breast cancer

**Fig. 7:**
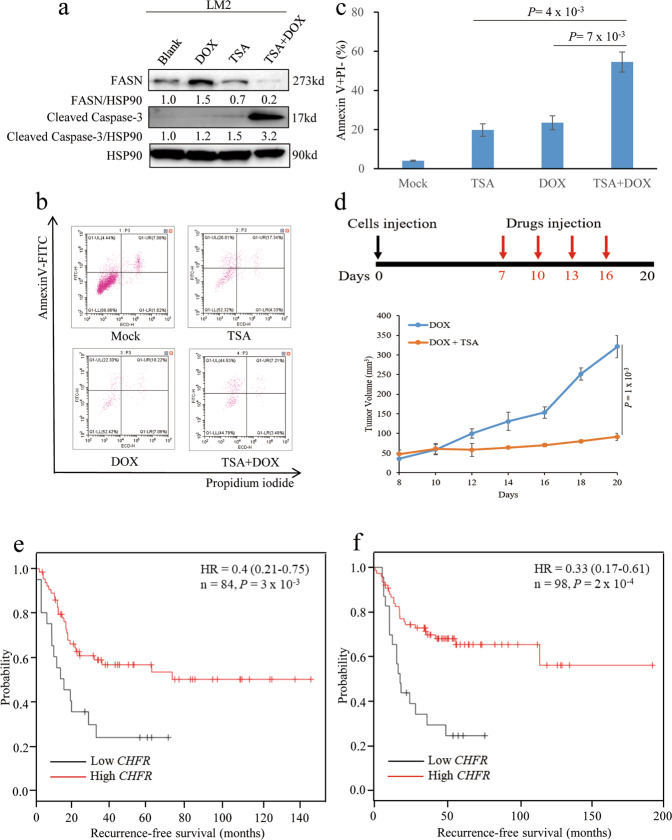
CHFR promotes apoptosis and correlates with good clinical outcome in malignant breast cancer. **a** LM2 cells were treated with or without DOX and TSA as indicated and immunoblotted with the FASN, Cleaved Caspase-3, and HSP90 antibodies. **b**, **c** SUM159 cells were treated as described in **a** and stained with propidium iodide and Annexin V-FITC and then analyzed by flow cytometry (**b**); **c** showed the percentage of early apoptotic (Annexin V+PI−; **c**) cells in SUM159 cells. **d** Tumor growth curves of nude mice with subcutaneous injection of LM2 cells. From day 7, mice received every 3 days intraperitoneal injections of doxorubicin with or without TSA. *n* = 3 mice per group. **e** Kaplan–Meier curves showing the distant relapse-free survival of patients with high or low expression of CHFR in the mesenchymal/basal subtype breast tumors. **f** Kaplan–Meier curves showing the distant relapse-free survival of patients with high or low expression of CHFR in the basal-like 1 subtype breast tumors. Significance of Mock versus DOX or TSA is shown. *n* = 3 wells per group. Data in **c**, **d** are the mean of biological replicates from a representative experiment, and error bars indicate s.e.m. Statistical significance was determined by a two-tailed, unpaired Student’s *t* test. The experiments were repeated three times. Statistical significance in **e**, **f** was determined by log-rank test.

Apoptosis has been established as a main cell death process linked to chemotherapy; to evaluate whether CHFR induces apoptosis, we treated LM2 cells with TSA individually or in combination with doxorubicin. Interestingly, either TSA alone or combined with doxorubicin could significantly downregulate FASN as described in Fig. 3. Moreover, co-treatment with TSA and doxorubicin drastically enhances the cleaved Caspase-3 expression level, which indicates that cells have undergone apoptosis (Fig. 7a). Indeed, Annexin V–propidium iodide staining by fluorescence-activated cell sorting analysis showed that co-treatment in LM2 cells increases early apoptosis by ten-fold compared to control group or three-fold compared to doxorubicin alone (Fig. 7b, c). To determine whether co-treatment has anticancer effects in vivo, we subcutaneously injected LM2 cells into the mice. Seven days after tumor cell implantation, we intraperitoneally injected doxorubicin with or without TSA into mice. At 3 weeks, the average tumor volumes in the TSA plus doxorubicin treatment group was 91.2 mm^3^, which was approximately 70% less than those in the doxorubicin treatment only group (321.2 mm^3^) (Fig. 7d). Cancer cells with therapy resistance including chemoresistance are likely to be a source of cancer recurrence and metastatic relapse [33]. To determine the correlation of CHFR expression with clinical outcome, we analyzed a cohort of human breast cancer patients in which transcriptomic profiling was obtained from 84 mesenchymal/basal subtype tumor samples [34]. This analysis revealed that patients with high CHFR expression levels (auto-select best cutoff) in their tumors had much better distant relapse-free survival than those with low CHFR expression levels (Fig. 7e; *P* = 3 × 10^−3^). In addition, analysis of 98 basal-like 1 subtype tumor samples also showed that high CHFR expression levels positively correlates with distant relapse-free survival of those patients [34] (Fig. 7f; *P* = 2 × 10^−4^). Moreover, we analyzed 177 mesenchymal subtype breast cancer patients with systematic treatment including conventional chemotherapy and found that low expression of CHFR confers mesenchymal breast cancers resistant to chemotherapy [34] (Fig. S6; *P* = 0.029). Collectively, these data suggest that downregulation of CHFR may contribute to overexpression of ZEB1 in human malignant breast tumors, which may lead to chemoresistance and eventually metastatic relapse.

## Discussion

TNBC (or basal-type breast cancer) remains the most challenging subtype of breast cancer to treat. Until now, therapies targeting specific molecular targets have rarely produced meaningful clinical improvements in the outcome of TNBC patients, and conventional chemotherapy is still the standard of treatment [35]. TNBC cells are highly heterogeneous populations [36], and different subpopulations of cells have their own characteristics, some may be more differentiated, but others exhibit CSC-like characteristics [37], which together contribute to the plasticity of tumor cells. Enhanced tumor cell plasticity has been shown to be an important driving force for tumor progression toward malignancy and recurrence of drug resistance [38–40]. Through this property of cellular plasticity, tumor cells constantly switch their status to adapt to the continuously changing tumor microenvironment and thus cause resistance to therapies. Recent findings suggest that ZEB1 is one of the most critical factors regulating tumor cell plasticity [5, 41]. Our previous results demonstrate that ZEB1 plays an important role in radiotherapy resistance in TNBC and that ZEB1 expression is dynamically distributed in different phases of the cell cycle [12]. Moreover, we found that cells in different cell cycle phases show different levels of resistance to conventional chemotherapeutic agents such as doxorubicin in this study, which is consistent with the expression level of ZEB1. Therefore, the identified underlined mechanism by which ZEB1 is dynamically regulated during cancer progression may explain why ZEB1-manipulated tumor plasticity/heterogeneity drive tumor cells to therapy resistance and relapse.

Although we and others found that phosphorylation, ubiquitination, and microRNAs could affect the ZEB1 protein level [12, 13, 23, 42], the molecules that involved in the dynamic regulation of ZEB1 stability are not known. By affinity purification followed by mass spectrometric analysis, we found that the cell cycle-regulated checkpoint ubiquitin E3 ligase, CHFR, may be an initiating factor in the dynamic regulation of ZEB1. CHFR has been conclusively shown to be an important regulator of cell cycle progression, with a dynamic distribution in different phases of the cell cycle that is exactly opposite to that of ZEB1. By overexpressing CHFR into cells, we found that CHFR acts as ubiquitin ligase of ZEB1, downregulates the protein stability of ZEB1 through the ubiquitin proteasome pathway. Furthermore, by overexpressing CHFR or knocking down ZEB1, we found that inhibition of the CHFR-ZEB1 signaling pathway significantly improved the therapeutic effects of clinical chemotherapeutic drugs such as 5-FU, paclitaxel, and doxorubicin on TNBC.

In this study, we presented a small-molecule inhibitor used to inactivate HDACs, TSA, that could specifically upregulate endogenous and ectopical expression of CHFR, which is histone acetylation independent. Previous study showed that 5-aza-dC alone, but not TSA, upregulate mRNA level of CHFR in colorectal cancer [15]; on the contrary, we found that only TSA could upregulate CHFR protein level in TNBC cells instead of mRNA level. The underlined mechanism warrants future investigation. Since ZEB1 is a transcriptional factor that is not easily druggable, indirectly inactivating ZEB1 by TSA open a new window for resistant TNBC. Notably, instead of directly affecting cyclinD1, CDK4/6, and BCL-XL [43], we found that co-treatment with TSA and chemotherapeutic drugs could synergistically inhibit cell viability by inducing apoptosis, which is clearly mediated by CHFR. In patients with breast cancer, CHFR was found to positively correlate with poor survival (both mesenchymal and basal-like 1 malignant breast cancer). Considering the low expression of CHFR in TNBC cells, restoration of CHFR protein expression in TNBC cells by TSA may provide new therapeutic strategies for the clinical treatment of TNBC.

## Materials and methods

### Cell culture

HEK293T, MDA-MB-231, MDA-MB-436, BT549, and MCF7 cell lines were from ATCC and cultured under conditions specified by the manufacturer. The SUM159 cell line was from S. Ethier (Medical University of South Carolina, USA) and cultured as described at http://www.asterand.com/Asterand/human_tissues/159PT.htm. The LM2 cells was a gift from Dr. Xiang Zhang (Baylor College of Medicine) and were cultured in Dulbecco’s Modified Eagle Medium supplemented with 10% fetal bovine serum and 1% penicillin/ streptomycin at 37 °C and 5% CO_2_.

### Plasmids including open reading frames (ORFs) and shRNAs

Human E3 ligase CHFR and the deletion mutants of CHFR were a gift from Xiaochun Yu (Michigan University, USA). CHFR shRNA (Clone ID: NM_018223.1-2974s1c1) construct was from Sigma. ZEB1 expression construct were from R. A. Weinberg. The ZEB1 shRNA was from Open Biosystems through MD Anderson’s shRNA core facility. The ZEB1 and CHFR ORFs were subcloned into the pBabe-SFB vector using the Gateway system (Invitrogen). ZEB1 mutants were constructed as described previously [23].

### RNA isolation and real-time PCR with reverse transcription

Total RNA was isolated using TRIzol reagent (Invitrogen) and then reverse transcribed with an iScript cDNA Synthesis Kit (Bio-Rad). The resulting cDNA was used for real-time PCR using the iTaq Universal SYBR Green Kit (Bio-Rad). β-Actin was used as an internal control. Real-time PCR and data collection were performed on a CFX96 instrument (Bio-Rad). The primer sequences for ZEB1: forward, 5’-AGAAGCCAGTGGTCATGATG-3’; reverse, 5’-CCTCAACAACCTCGTGGAAGCATAC-3’, the primer sequences for β-actin: forward, 5’-TCCCTGGAGAAGAGGCTACGA-3’; reverse, 5’-AGGAAGGAAGGCTGGAAGAG-3’, and the primer sequences for CHFR: 5’-GAGGTAAAGCGTTTATAGCC-3’; reverse, 5’-TGCCTTCTGTACTCAGGACACTGCC-3’. All quantitative PCR reactions were performed in triplicate.

### Lentiviral transduction

The production of lentivirus and infection of target cells were performed as described previously [12]. Lentiviral supernatant was collected at 36, 60, and 84 h after co-transfection of psPAX2, pMD2.G, and the shRNA- or ORF-containing vector into HEK293T cells and was added to the target cells. Forty-eight hours later, the infected cells were selected with 1 μg ml^−1^ for puromycin.

### Immunoblotting

Western blot analysis was performed with sodium dodecyl sulfate-polyacrylamide gel electrophoresis (SDS-PAGE) gels using standard methods. Briefly, cultured cells were lysed in RIPA buffer containing protease inhibitors (Roche) [12]. Proteins were separated by SDS-PAGE and blotted onto a nitrocellulose membrane (Bio-Rad). Membranes were probed with the specific primary antibodies, followed by secondary antibodies. The bands were visualized by chemiluminescence (Millipore). The following antibodies were used: antibodies against ZEB1 (1:1000, Bethyl Laboratories, A301-922A), CHFR (1:2000, ABclonal, A10447), FASN (1:1000, Cell Signaling Technology, 3180S), Acetyl-Histone H3 (Lys27) (1:1000, Cell Signaling Technology, 8173S), cyclin A (1:1000, Santa Cruz, sc-751 clone H-432), p-H3 (1:1000, S10, Cell Signaling Technology, 9701), HA (1:2500, Santa Cruz, sc-7392, Clone F-7), FLAG (1:5000, Sigma, F3165, clone M2), MYC (1:5000, Santa Cruz, SC-40, clone 9E10), β-actin (1:2000, Santa Cruz, SC-47778), HSP90 (1:3000, BD Transduction Laboratories, 610419, clone 68), and GAPDH (1:3000, Thermo, MA515738, clone GA1R) at 4 °C overnight, followed by incubation with goat anti-rabbit IgG or goat anti-mouse IgG (1:3000, Boster) at room temperature for 1 h. The Image J program (http://rsbweb.nih.gov/ij/download.html) was used for densitometric analysis of western blots, and the quantification results were normalized to the loading control.

### Immunoprecipitation and pulldown assays

Cells were lysed in NETN buffer (100 mM NaCl, 1 mM EDTA, 20 mM Tris-HCl (pH 8.0), 0.5% Nonidet P-40) containing protease inhibitors (Roche). For pulldown of SFB-tagged proteins or MYC-tagged proteins, cell extracts were incubated with streptavidin–Sepharose beads (Amersham Biosciences) or Anti-Myc magnetic beads (Thermo Fisher, 88842) at 4 °C for 2 h. The beads was washed with NETN buffer and the bound proteins were eluted by boiling in 1× Laemmli buffer.

### Chemicals

The chemicals used for treating cells are TSA (Selleckchem, S1045), SAHA (Sigma, SML0061), MG132 (Santa Cruz Biotechnology, sc-201270), CHX (Sigma, C7698), doxorubicin (Sigma, D1515), 5-FU (Sigma, F6627), and paclitaxel (Sigma, T7191).

### Tandem affinity purification and mass spectrometry

293T cells were transfected with SFB-tagged ZEB1. Six hours before collection, 10 µM MG132 was added into cells. The expression of exogenous protein was confirmed by immunoblotting. Affinity purification and mass spectrometric analysis were performed as described previously [12].

### In vivo ubiquitination assays

For the in vivo ubiquitination assay, HEK293T cells were harvested 48 h after transfection with the indicated plasmids. For denaturing, lysates were heated at 95 °C for 5 min in the presence of 1% SDS, followed by 10-fold dilution with lysis buffer (to 0.1% SDS) and sonication, as described previously [44]. The cell lysates were incubated with S-protein agarose (Millipore) for 2 h, and then the beads were washed with lysis buffer three times and subjected to immunoblotting analysis.

### Cell viability assay

The indicated TNBC cells were seeded in 96-well plates (10,000 cells per well) and treated with or without chemotherapeutic drugs. Cells were then fixed at the indicated times and stained with crystal violet (0.05% w/v in formalin). The dye from stained cells was dissolved in 10% acetic acid and the absorbance was measured at 570 nm.

### Identification of differentially expressed genes

TNBC patients was obtained from the TCGA data portal (https://tcga-data.nci.nih.gov/tcga/), and RNA expression for TNBC was obtained using TCGA-BRCA.GDC_phenotype (August 8, 2020). We used R studio for analysis to get mesenchymal stem-like clinical data and removed the adjacent and abnormal data. Differential gene expression analysis was performed to assess the function of genes.

### Molecular docking

The three-dimensional structure of the proteins were downloaded from the RCSB protein database (www.rcsb.org). The proteins used (PDB ID: 1lgp, 2xp0, and 2e19) were opened in Autodock Tools 1.5.6. By adding all hydrogen atoms, the Gasteiger particle size was calculated and the non-polarity was combined. After hydrogen, we define it as a receptor and save it as a pdbqt file. Small-molecule drugs were downloaded from the National Library of Medicine (https://pubchem.ncbi.nlm.nih.gov/). The drugs used (PubChem CID: 444732, 5311) were opened in Autodock Tools 1.5.6. The molecular docking simulation has been performed by Autodock 4.2 package, and the corresponding Autodock Tools 1.5.6 has been used to prepare all necessary input files and analyze the docking results.

### Mice

All mice used in this study were supplied by and housed in the Research Animal Support Facility at Huazhong University of Science & Technology. Six-week-old female athymic nude mice were used for subcutaneous injection of 5 × 10^6^ LM2 human breast cancer cells. The tumor volume was calculated according to the equation *v* = length × width^2^ × 1/2. For drug treatment experiments, 7 days after tumor cell injection, nude mice were treated with 4.5 × 10^−3^ mg kg^−1^ doxorubicin (APExBio, 3966) with or without 9 × 10^−3^ mg kg^−1^ TSA (APExBio, A8183) through intraperitoneal injection every 3 days until the endpoint, as indicated. All animal experiments were performed in accordance with a protocol approved by the Institutional Animal Care and Use Committee of Tongji Medical College, Huazhong University of Science & Technology.

### Patients’ survival curve

Kaplan–Meier plots were generated to illustrate the relationship between patients’ disease-free survival and gene expression levels of CHFR or ZEB1. Data were obtained from http://kmplot.com/analysis/. Auto-select best cutoff was used in the analysis. The probe used in Figs. 7d, e and S6: 218803_at; the probe used in Fig. S1a, d: 210875_at; the probe used in Fig. S1b: 239952_at; the probe used in Fig. S1c: 212764_at. The relationship was tested by log-rank test.

### Statistics and reproducibility

Each experiment was repeated three times or more. Unless otherwise noted, data are presented as mean ± s.e.m., and Student’s *t* test (unpaired, two-tailed) was used to compare two groups of independent samples. The data analyzed by *t* test meet normal distribution; we used an *F*-test to compare variances, and the variances are not significantly different. Therefore, when using an unpaired *t* test, we assumed equal variance, and no data points were excluded from the analysis. *P* < 0.05 was considered statistically significant.

## Supplementary information

CHFR regulates chemoresistance in triple negative breast cancer through destabilizing ZEB1

Table S1

## Data Availability

The datasets and materials used and/or analyzed during the current study are available from the corresponding author on reasonable request.
