# Supplementary material for: CHFR regulates chemoresistance in triple-negative breast cancer through destabilizing ZEB1
Source: Cell Death Dis. 2021 Aug 30;12(9):820. doi: 10.1038/s41419-021-04114-8 (PMC8405615; doi:10.1038/s41419-021-04114-8)
Supplement: Supplementary file 1 — CHFR regulates chemoresistance in triple negative breast cancer through destabilizing ZEB1 [file 41419_2021_4114_MOESM1_ESM.docx]

SUPPLEMENTARY INFORMATION

CHFR regulates chemoresistance in triple negative breast cancer through destabilizing ZEB1

Hong Luo, Zhicheng Zhou, Shan Huang, Mengru Ma, Manyu Zhao, Lixu Tang, Yuan Quan, Yiming Zeng, Li Su, Jongchan Kim, Peijing Zhang

*Corresponding author: [yuanquan1011@gmail.com](mailto:yuanquan1011@gmail.com), [zhangpeijing@hust.edu.cn](mailto:zhangpeijing@hust.edu.cn)


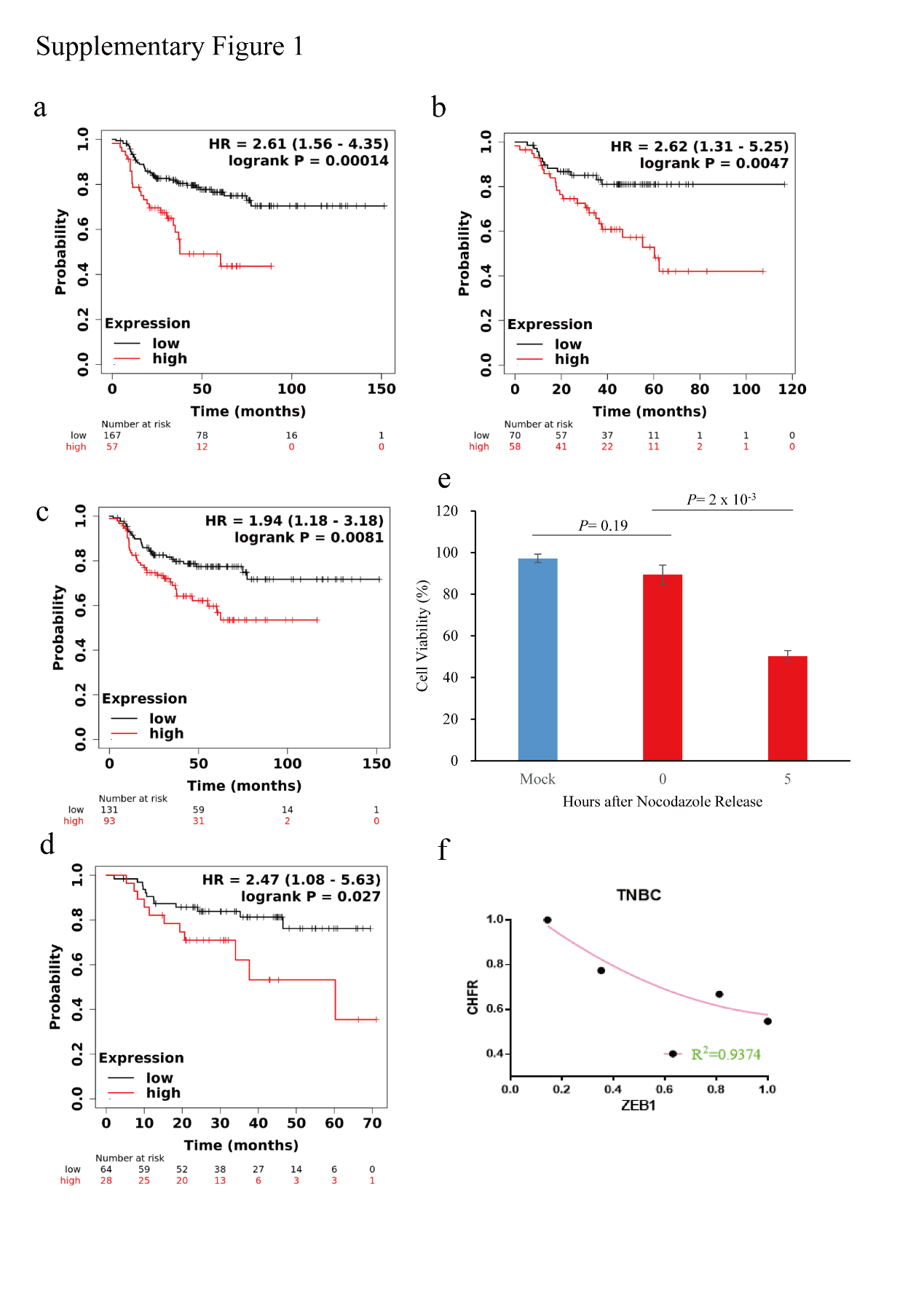


**Supplementary figure 1 ZEB1 correlates with CHFR and poor clinical outcomes in human breast cancer**

**a-d** Kaplan–Meier curves showing the distant relapse-free survival of patients with high or low expression of ZEB1 in the basal subtype breast tumors treated with chemotherapy (a-c) or neoadjuvant therapy (d). **e** SUM159 cells were treated with or without 0.5 µgml^-1^ nocodazole overnight, the mitotic cells were "shaken off" and then released into normal medium. 0 and 5 hours after releasing, cells were treated without (mock) or with DOX (0, 5 hours) for 36 hours, and then cell viability was analysed. **f** RNA-seq expression data of breast cancer was downloaded from TCGA (gdc_download_20200801_135953. 599013), screened out triple-negative breast cancer samples with RStudio-1.3.1056, compared the expression differences between cancer and normal tissues, randomly selected 4 samples of TNBC (A total of 6 samples fully meet the requirements, one is repeated and one is completely outlier) mesenchymal/basal subtype patient samples, and analysed correlation between CHFR and ZEB1. Significance of Mock versus DOX is shown. n = 3 wells per group. Data in f is the mean of biological replicates from a representative experiment, and error bars indicate s.e.m. Statistical significance was determined by a two-tailed, unpaired Student’s t-test. The experiments were repeated 3 times. Statistical significance in a-d and e was determined by a log-rank test.The p value in f was calculated from a linear regression analysis. R is the correlation coefficient.


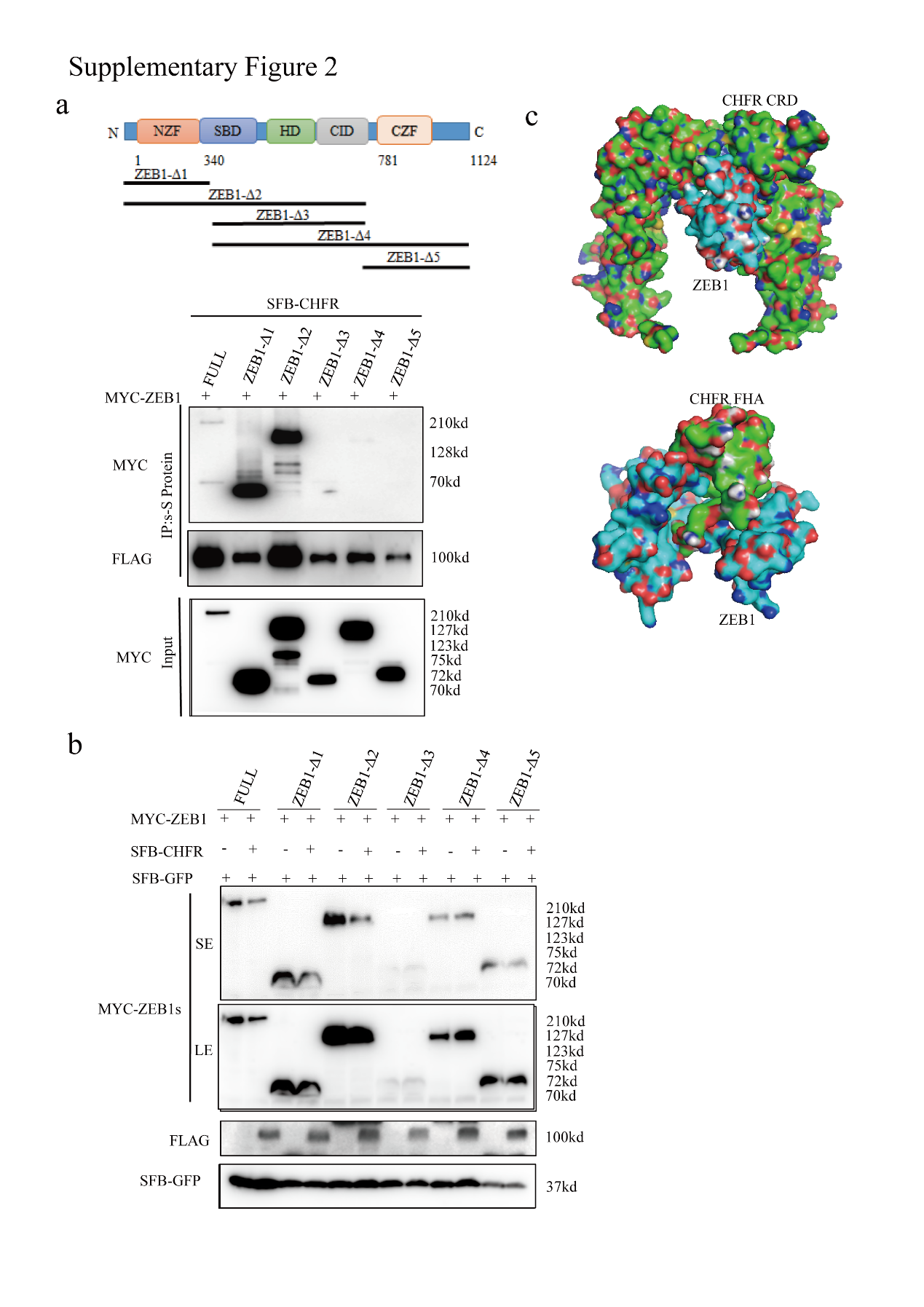


**Supplementary figure 2 Interaction and regulation of ZEB1 and its mutants and/by CHFR protein**

**a** Schematic diagram of the domains and mutants of ZEB1. Transfected full length MYC-ZEB1, MYC-ZEB1Δ1, MYC-ZEB1Δ2, MYC-ZEB1Δ3, MYC-ZEB1Δ4, MYC-ZEB1Δ5 and SFB-CHFR into HEK293T cells, cells were pulled down with s-S protein beads, and immunoblotting with the MYC and FLAG antibodies. SE: short exposure; LE: long exposure. **b** HEK293T cells were transfected as described in (a), then collected, and immunoblotting with the MYC and FLAG antibodies. **c** Download crystal structure of the ZEB1 (PDB ID: 2E19), CHFR-FHA (PDB ID: 1LGP) or CHFR-CRD (PDB ID: 2XP0) domains from RCSB Protein Data Bank (www.rcsb.org). They were added all hydrogen atoms, calculating Gasteiger charges, and merging non-polar hydrogens, then defined them as acceptors and saved them as pdbqt files by AutodockTools1.5.6. Molecular docking analyses were performed by AutoDock Vina (ver. 1.1.2) which is one of the most widely used methods for protein-ligand docking.


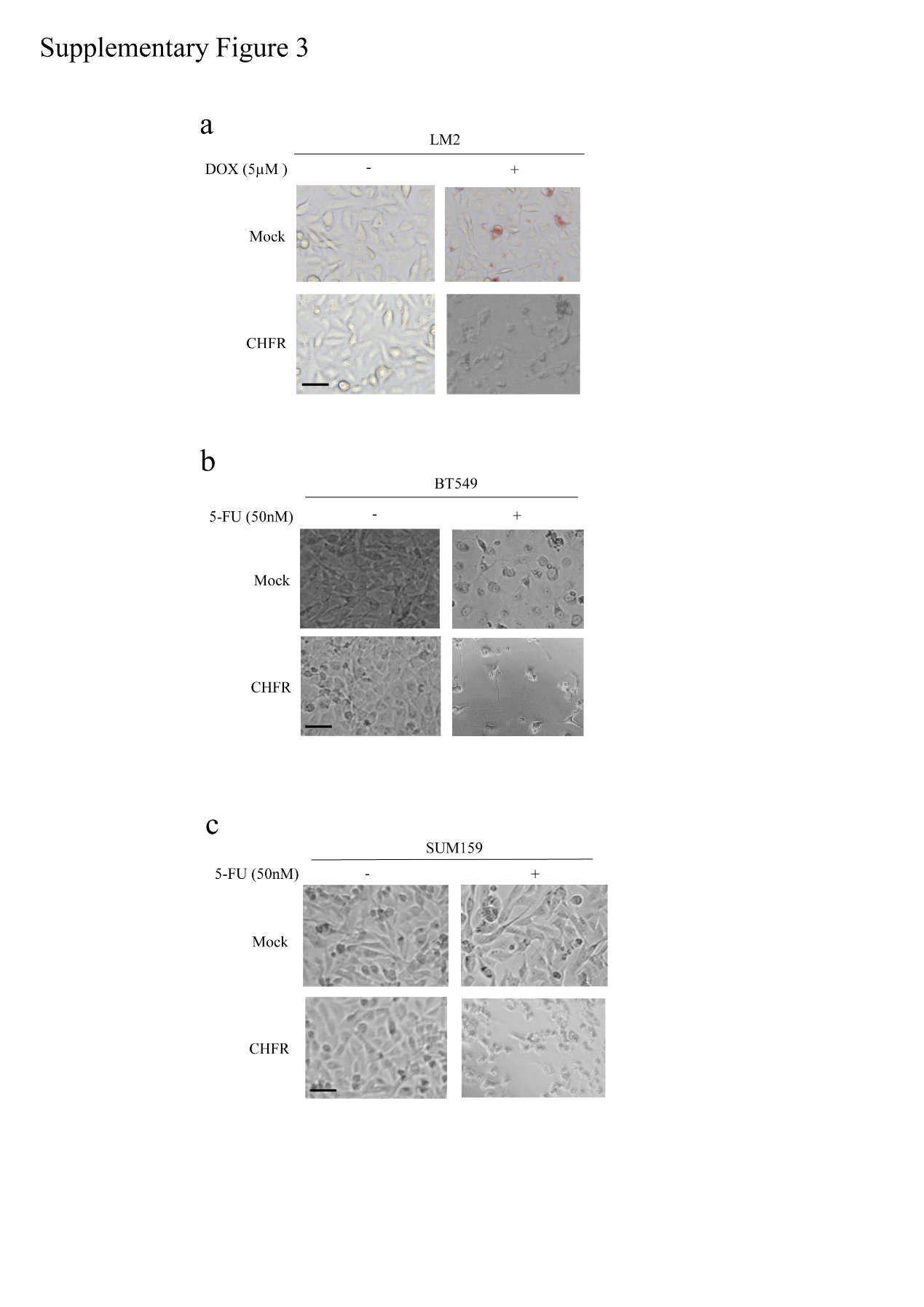


**Supplementary Figure 3 CHFR sensitizing TNBC to chemotherapy drugs.**

**a** Phase contrast images of LM2 cells stably transfected mock or CHFR, and treated with DOX for 36 hours, and then photographed with a biological microscope. **b** Phase contrast images of BT549 cells stably transfected mock or CHFR, and treated with 5-FU for 36 hours. **c** Phase contrast images of SUM159 cells stably transfected mock or CHFR, and treated with 5-FU for 36 hours. Scale bars = 50µm.

**
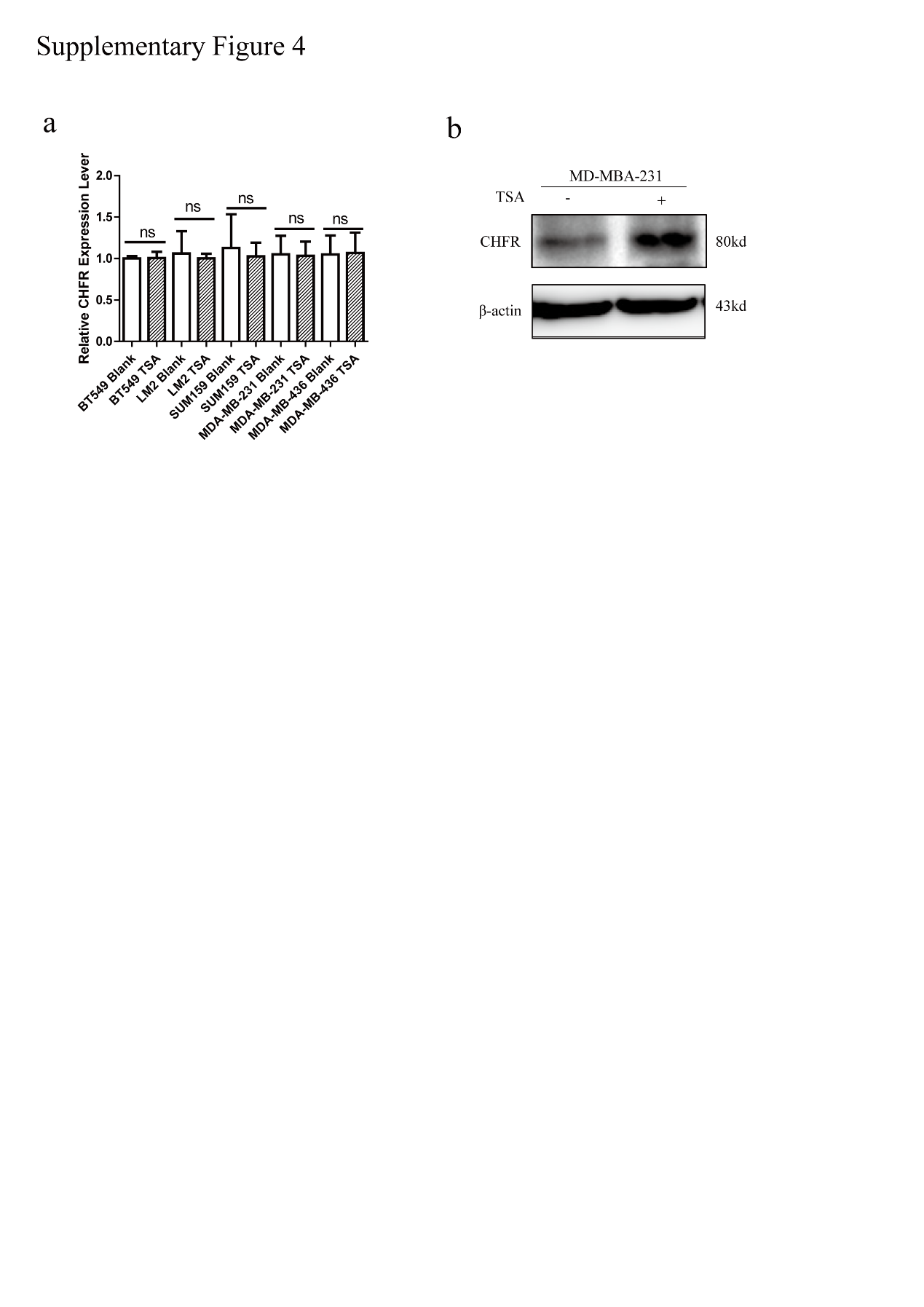
**

**Supplementary Figure 4 TSA treatment does increase the protein level of CHFR, but not mRNA level**

**a** TSA was added to LM2, SUM159, MDA-MB-231 and MDA-MB-436 cells and cultured for 36 hours. After RNA was extracted, the expression of CHFR mRNA was detected by qPCR. **b** TSA was added to MDA-MB-231 and cultured for 36 hours, and immunoblotting with the CHFR and β-ACTIN antibodies. Significance of blank versus TSA is shown. n = 3 wells per group. Data in a is the mean of biological replicates from a representative experiment, and error bars indicate s.e.m. Statistical significance was determined by a two-tailed, unpaired Student’s t-test.


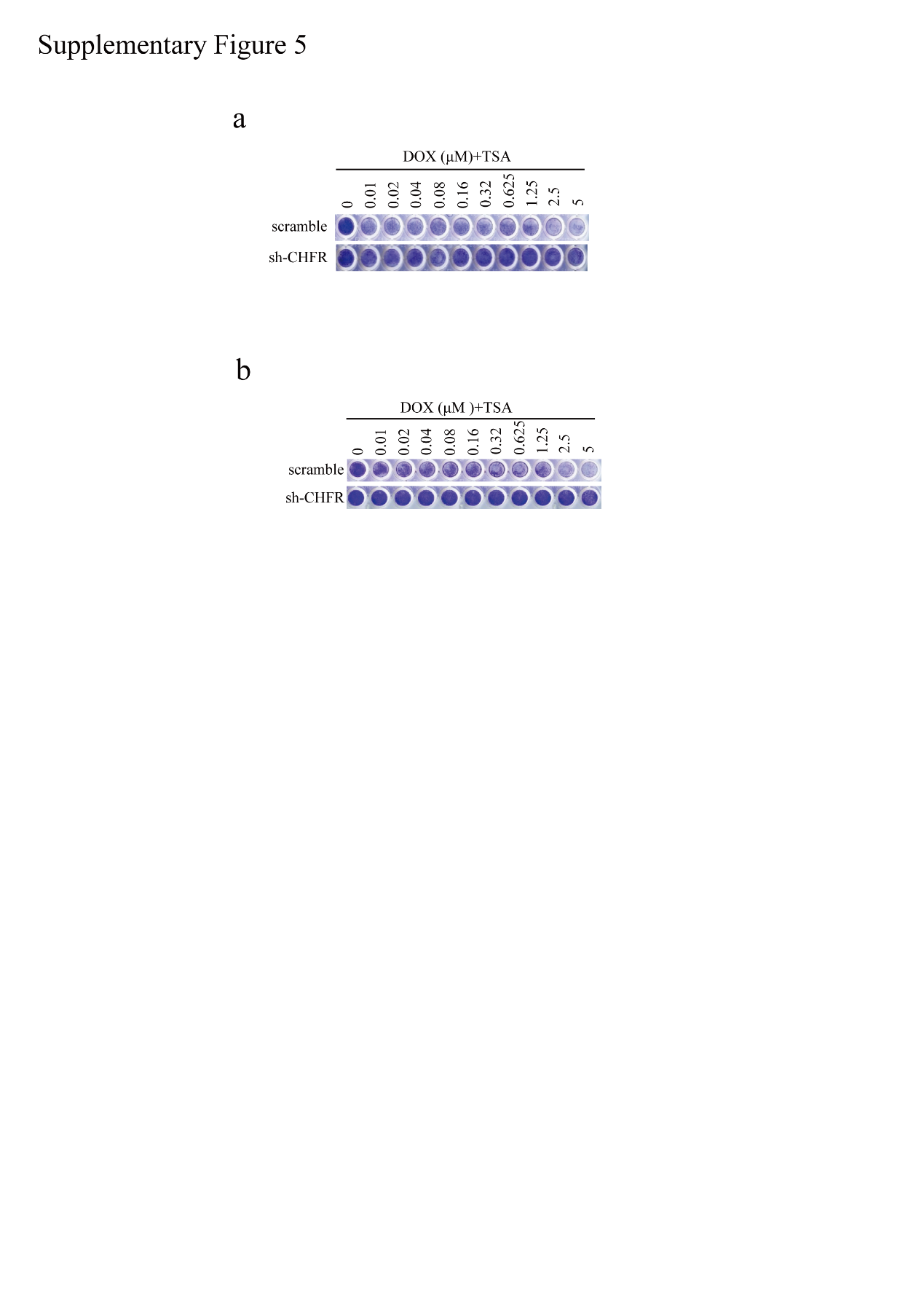


**Supplementary Figure 5 Adding TSA and DOX chemotherapeutics to stable cells knockdown of CHFR**

**a** LM2 cells stably knocked down CHFR were co-treated with TSA and DOX as indicated for 36 hours. Then fixed with 10% methanol, and then stained with 1:1000 crystal violet. **b** MDA-MB-231 stably knocked down CHFR were co-treated with TSA and DOX as indicated for 36 hours. Then fixed with 10% methanol, and then stained with 1:1000 crystal violet.


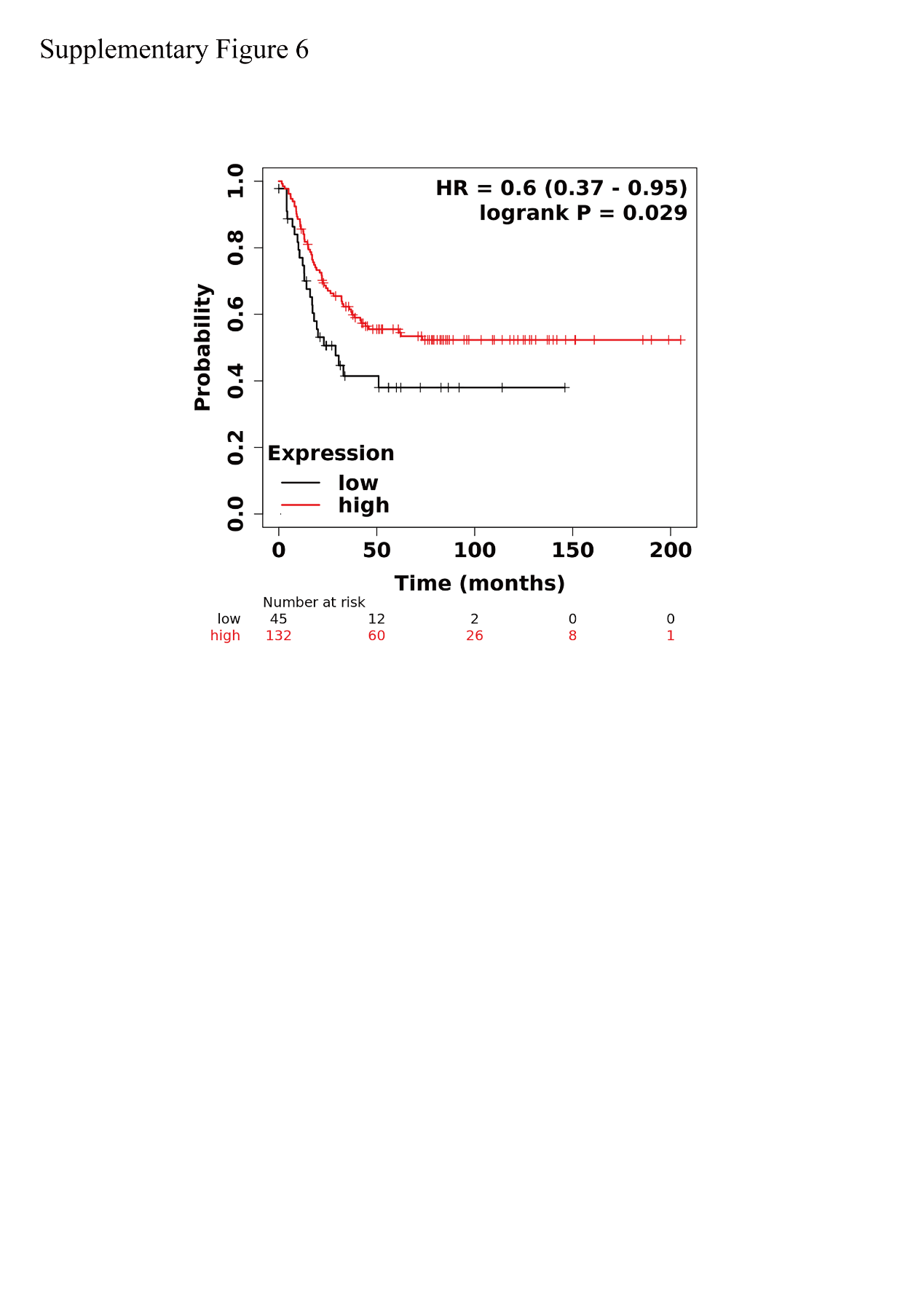


**Supplementary figure 6 CHFR correlates with good clinical outcome in human mesenchymal breast cancer treated with chemotherapy**

Kaplan–Meier curves showing the distant relapse-free survival of patients with high or low expression of CHFR in the mesenchymal subtype breast tumors treated with chemotherapy. Statistical significance was determined by a log-rank test.
