# Supplementary material for: CHFR regulates chemoresistance in triple-negative breast cancer through destabilizing ZEB1
Source: Cell Death Dis. 2021 Aug 30;12(9):820. doi: 10.1038/s41419-021-04114-8 (PMC8405615; doi:10.1038/s41419-021-04114-8)
Supplement: Supplementary file 2 — Table S1 [file 41419_2021_4114_MOESM2_ESM.docx]

Table S1 List of ZEB1-interacting proteins identified by TAP-MS analysis

Peptide hits Protein Name

53 ZEB1

46 HSPA9

45 HSPA8

36 HSPA5

24 HSPA4

22 C15orf44

21 HSPA1A

21 RPS3A

19 C12orf11

18 CTBP2

17 RPS3

17 DHX9

15 TUBB2C

15 DDX21

13 HSPA1L

13 INTS10

13 RPS4X

12 CTBP1

12 C12orf11

12 HNRNPM

11 SIRT1

11 HNRNPU

11 RPS9

11 NCL

11 RPL3

10 NPM1

10 RPLP0

9 RPL4

9 PARP1

8 CHFR

8 RPL7

8 SLC25A5

8 IPI

8 RPS18

8 RPS16

7 XRCC6

7 IGF2BP1

7 RPL6

7 DDX1

7 HSPH1

7 DDX5

6 YBX1

6 SYNCRIP

6 PTCD3

6 RPS13

6 CKAP4

6 TUBA4A

6 PRKDC

6 C22orf28

6 RPS8

6 HNRNPR

6 ILF3

6 RPL23A

6 RPS2

6 PRDX1

5 CSDA

5 TCP1

5 RPS15A

5 TP53

5 RPL7A

5 RPS14

5 CAD

4 ILF2

4 HNRNPF

4 DAP3

4 PPP2R1A

4 SSB

4 TUFM

4 AHCYL2

4 EEF1A2

4 SLC25A3

4 DDX17

4 XRCC5

3 C7orf26

3 NAP1L1

3 USP7

3 RNH1

3 SLC25A6

3 MRPS35

3 MRPS27

3 C1QBP

3 AHCYL1

3 IPO8

3 AIFM1

3 PABPC1

3 EMD

2 GNL3

2 C14orf166

2 G3BP2

2 KDM1

2 UBE2O

2 CSNK2A1

2 GTPBP1

2 H1FX

2 ZNF516

2 SLC25A13

2 PPM1G

1 RPL14

1 RPS6

1 G3BP1

1 E4F1

1 SERBP1

1 RPLP1
